# Detection of *Candidatus* Liberibacter asiaticus and five viruses in individual Asian citrus psyllid in China

**DOI:** 10.3389/fpls.2024.1357163

**Published:** 2024-02-06

**Authors:** Luqin Liu, Jing Chen, Junyao Jiang, Jiamei Liang, Yaqin Song, Qi Chen, Fuling Yan, Ziqin Bai, Zhen Song, Jinxiang Liu

**Affiliations:** ^1^ Citrus Research Institute, Southwest University/National Citrus Engineering Research Center, Chongqing, China; ^2^ Guangxi Academy of Specialty Crops, Guangxi Citrus Breeding and Cultivation Research Center of Engineering Technology, Guangxi, China; ^3^ Fruit Research Institute, Guizhou Provincial Academy of Agricultural Sciences, Guizhou, China

**Keywords:** Asian citrus psyllid, *Candidatus* Liberibacter asiaticus, insect viruses, Huanglongbing, co-infection

## Abstract

**Introduction:**

Asian citrus psyllid (ACP, *Diaphorina citri*) is an important transmission vector of “*Candidatus* Liberibacter asiaticus” (*C*Las), the causal agent of Huanglongbing (HLB), the most destructive citrus disease in the world. As there are currently no HLB-resistant rootstocks or varieties, the control of ACP is an important way to prevent HLB. Some viruses of insect vectors can be used as genetically engineered materials to control insect vectors.

**Methods:**

To gain knowledge on viruses in ACP in China, the prevalence of five RNA and DNA viruses was successfully determined by optimizing reverse transcription polymerase chain reaction (RT-PCR) in individual adult ACPs. The five ACP-associated viruses were identified as follows: diaphorina citri bunyavirus 2, which was newly identified by high-throughput sequencing in our lab, diaphorina citri reovirus (DcRV), diaphorina citri picorna-like virus (DcPLV), diaphorina citri bunyavirus (DcBV), and diaphorina citri densovirus-like virus (DcDV).

**Results:**

DcPLV was the most prevalent and widespread ACP-associated virus, followed by DcBV, and it was detected in more than 50% of all samples tested. DcPLV was also demonstrated to propagate vertically and found more in salivary glands among different tissues. Approximately 60% of all adult insect samples were co-infected with more than one insect pathogen, including the five ACP-associated viruses and *C*Las.

**Discussion:**

This is the first time these viruses, including the newly identified ACP-associated virus, have been detected in individual adult ACPs from natural populations in China’s five major citrus-producing provinces. These results provide valuable information about the prevalence of ACP-associated viruses in China, some of which have the potential to be used as biocontrol agents. In addition, analysis of the change in prevalence of pathogens in a single insect vector is the basis for understanding the interactions between *C*Las, ACP, and insect viruses.

## 1 Introduction

Citrus is the most widely cultivated fruit tree in southern China and is an important economic fruit (Zhou, 2018). Moreover, China leads the world’s production with its yield amounting to 44.6 million metric tons and accounting for 28% of the global output in summer 2021 and winter 2021/22 citrus seasons according to the World Citrus Organization (WCO, https://worldcitrusorganisation.org/). However, Huanglongbing (HLB) is a threat to citrus production and causes premature fruit drop, resulting in a 30-100% yield reduction and producing the most severe economic losses to the citrus industry (Zhou, 2020; Chinyukwi et al., 2023). *C*andidatus Liberibacter asiaticus (*C*Las) is the bacterial pathogen causing HLB. Citrus trees affected by *C*Las have reduced water absorption and accumulation of nutrients, which may cause plant death (Atta et al., 2020). While Asian citrus psyllid (ACP) is an insect vector that transmits *C*Las, it is also an important factor in the prevalence of HLB (Vyas et al., 2015; Snyder et al., 2022). At present, effective methods for the prevention and control of HLB have not been found (Ghosh et al., 2022). The prevention and control of its vector, ACP, is one of the most important measures to prevent HLB epidemics. ACP population control relies heavily on insecticides, but chemical strategies lead to the development of chemical resistance among ACP populations, pollute the environment, and may have negative effects on beneficial organisms (Leong et al., 2022). To avoid the development of insecticide-resistant insect populations, biological control strategies are being considered.

Viruses, as the most abundant microorganisms on Earth, are present in all groups of organisms (Weitz and Wilhelm, 2012). With the development of metagenomics, viruses from families, including *Baculoviridae*, *Parvoviridae*, *Flaviviridae*, and *Bunyaviridae*, are being increasingly discovered (Simmonds et al., 2017). Some viruses have complex relationships with their insect hosts, affecting the growth and development of insects or helping to spread pathogens (Bolling et al., 2015; Marklewitz et al., 2015). Marutani-Hert et al. (2009) discovered two diaphorina citri reovirus (DcRV) sequences in ACP through high-throughput sequencing. Nouri et al. (2016) discovered diaphorina citri picorna-like virus (DcPLV), diaphorina citri bunyavirus (DcBV), diaphorina citri associated C virus (DcACV), and a DNA virus called diaphorina citri densovirus (DcDV). According to phylogenetic analysis, DcPLV is close to the *Iflaviridae* based on the RNA-dependent RNA polymerase (RdRp); DcRV is closely related to *Fijivirus*; DcDV placed closer to the viruses from the genus *Iteradensovirus* based on NS2 amino acid; DcBV was most closely to *Phasmaviridae* (Nouri et al., 2016; Chen et al., 2020). Britt et al. (2020) found diaphoerina citri flavi-like virus (DcFLV) and another reovirus, diaphorina citri cimodo-like virus (DcCLV), and detected the prevalence of these viruses in Florida in 5 ACPs as a group sample. They found that DcACV had the highest detection rate in ACPs in Florida. However, it is unclear which virus is the most prevalent in ACPs in citrus groves in China.

Interactions between viral and bacterial pathogens in insects have been reported. Insect symbiotic bacterium *Sulcia* harbors a viral pathogen (rice dwarf virus) and mediates its transovarial transmission to offspring in leafhoppers (Jia et al., 2017). Teixeira et al. (2008) reported that the bacterial symbiont *Wolbachia* renders *Drosophila melanogaster* more resistant to the Drosophila C virus, reducing the viral load of infected flies. The effects of *C*Las infection on the metagenome of the *Diaphorina citri* gut endosymbiont were recently reported (Pan et al., 2023). Moreover, the titer of *Wolbachia* has been demonstrated to have a positive correlation with the *C*Las titer (Fagen et al., 2012). However, little is known about the relationship between *C*Las and ACP-associated viruses. Whether these insect viruses also have similar effects with *C*Las in ACP needs to be further explored.

In the present study, the main objectives were to determine the prevalence of the newly identified diaphorina citri bunyavirus 2 (DcBV2) which also belongs to *Bunyavirales* as well as DcBV (unpublished data) and four previously identified ACP-associated viruses, namely DcRV, DcPLV, DcBV, and DcDV, in individual adult ACPs from citrus groves in China, using PCR-based methods and to evaluate the relationship between *C*Las and ACP-associated viruses. Adult ACP populations were collected from five of China’s major citrus-producing and ACP-inhabiting regions: Guizhou, Jiangxi, Guangdong, Guangxi, and Sichuan. This study will provide foundational knowledge of the above viruses with regard to their infection rate in ACP in five major citrus-producing and ACP-inhabiting provinces.

## 2 Materials and methods

### 2.1 Materials

A total of 551 ACPs were gathered in Guizhou, Jiangxi, Guangdong, Guangxi, and Sichuan from 2019 to 2023 during the outbreak (**Supplementary Table S1**). ACP samples were collected randomly from semi-unmanaged citrus orchards by manual aspiration with flukes. The fluke is made of leather tube, mesh yarn and suction head. Immediately after collection, they were stored in 50 ml centrifuge tubes and transported to the Citrus Seedling Detoxification Center Laboratory of the Citrus Research Institute of Southwest University on dry ice and stored at −80°C for later use.

### 2.2 Total RNA extraction

Each ACP was removed and placed in a 1.5 mL centrifuge tube. A volume of 200 μL of Trizol reagent (Invitrogen, Beijing, China), and three 1.5 mm steel balls were added to the tube. These were then ground at 50 Hz for 2 min in a high-throughput tissue grinder (SCIENTZ-48L, Ningbo, China) at −20°C. Total RNA of individual adult ACPs was extracted according to the Trizol reagent instruction manual (Invitrogen, Beijing, China). RNA samples were stored at −20°C for later use.

### 2.3 RT-PCR detection of ACP virus

Using the total RNA of individual ACPs as a template, the primers shown in **Table 1** were used for RT-PCR to detect DcRV, DcPLV, DcBV, DcDV, *C*Las, and DcBV2. *β-Actin* of ACP was used as the internal reference gene to verify successful RNA extraction. RT-PCR was carried out in a 10 μL reaction mixture containing 5 μL of 2×1 Step Buffer, 0.4 μL of the forward primer (10 μM), 0.4 μL of the reverse primer (10 μM), 0.4 μL of PrimeScript 1 Step Enzyme Mix (Takara, Sichuan, China), 2.8 μL RNase Free dH2O, and 1 μL RNA. RT-PCR cycling parameters were as follows: 50°C for 30 min, 94°C for 2 min, and 35 cycles of denaturation at 94°C for 30 s, extension at 55°C for 30 s, and 72°C for 1 min. The RT-PCR products were separated by 1.2% agarose gel electrophoresis and purified using the Easy Pure^®^ Quick Gel Extraction Kit (Transgene, EG101-02). The purified products were connected to the pGEM-T Easy vector (Promega, Beijing, China) and transferred to *Escherichia coli* DH5α (WeiDi, Shanghai, China). The positive clones were sent to Qingke or BGI Biotechnology Co., Ltd. for sequencing. The sequencing results of the positive clones of each fragment were compared with sequences recorded in the NCBI database.

**Table 1:** List of primers used to detect *C*Las and 5 Asian citrus psyllid (ACP)-associated viruses.

Viruses	Primers	Tm	Product size
DcRV	F: 5’ TTTTCCCAGGTACATCGA 3’ R: 5’ ACCATTCAGCCAGTCCTA 3’	54°C	900 bp^1^
DcPLV	F: 5’ TAGGTGAACGTGATAATCCTGGTAT 3’ R: 5’ CAGAACGTCTGTTATGAATCGGAC 3’	56°C	698 bp^1^
DcBV	F: 5’ CTATGAAGGCAGGAACAGAGACAA 3’ R: 5’ GTTTCATCCCCACCTTCCACTAA 3’	58°C	624 bp^2^
DcDV	F: 5’ AGTCGGTGAGACTGATATCTTCGAGACC 3’ R: 5’ GTTTAGTTCGCTTGTCGGTTACACAGG 3’	61°C	1068 bp^1^
*C*Las	F: 5’ GCGCGTATGCAATACGAGCGGCA 3’ R: 5’ GCCTCGCGACTTCGCAACCCAT 3’	60°C	1160 bp^3^
DcBV2	F: 5’ GCTGAAAGAAAGTAAAGGAGTG 3’ R: 5’ GCAGCCTACGGATTTGAGGTG 3’	55°C	905 bp^4^
*β-Actin*	F: 5’ CCCTGGACTTTGAACAGGAA 3’ R: 5’ CTCGTGGATACCGCAAGATT 3’	57°C	170 bp^5^

^1^ Britt et al., 2020; ^2^Accession number: PP025819; ^3^ Jagoueix et al, 1994; ^4^Accession number: PP025816; ^5^ Liu et al., 2020; CLas, Candidatus Liberibacter asiaticus;diaphorina citri reovirus, DcRV; diaphorina citri picorna-like virus, DcPLV; diaphorina citri bunyavirus, DcBV; diaphorina citri densovirus-like virus, DcDV; diaphorina citri bunyavirus 2, DcBV2; F: Forward Primer; R: Reverse Primer; bp: base pair.

### 2.4 Collection of ACPs’ offspring

The nascent male and female ACPs were reared on the new shoots of clean orange jessamine and covered with gauze ribbons to prevent ACPs from escaping. After the male and female ACPs mated and laid eggs, we removed the ACP parents and left the offspring to grow. Then when ACP generation had developed to 5th instar nymph, they were individually taken out to determine whether they were infected with DcPLV using RT-PCR as methods shown in 2.2.

### 2.5 RT-qPCR for DcPLV detection

We collected 80 adult ACPs, dissected them with forceps in 0.1 M phosphate-buffered saline (PBS, pH 7.2) under a stereo microscope (Leica, Wetzlar, Germany) and collected guts, salivary glands, testes, ovaries, Malpighian tubules, and remnant tissues. RNA was extracted from each tissue with 4 biological replicates per sample. The concentration of RNA isolated from various organs of ACP was shown in **Supplementary Table S2**.

First-strand cDNA was synthesized using an All-In-One 5×RT MasterMix kit (abm, Chongqing, China) according to the manufacturer’s instructions. RNA isolated from various organs of the insect was added to 20 µl reactions with 1 µg as a template for reverse transcription. Quantification PCR was performed in 20 µL reactions with 100 ng cDNA using a BlasTaq™ 2×qPCR MasterMix kit (abm, Chongqing, China) and primers (forward primer: AAACAGTGGCGAGGAACGAT, reverse primer: CCACCAAATCCGGTCTGTCA) at concentrations of 0.25 μM according to the manufacturer’s instructions in the CFX96 touch system (Bio-Rad, Berkeley, California, USA). *β-Actin* of ACP was selected to normalize the DcPLV-expression level.

### 2.6 Statistical analysis

All detection data were calculated using Excel software. Quantitative analyses for the relative levels of gene accumulation were analyzed according to the 2^−ΔΔCt method. One-way analysis of variance (ANOVA) followed by the Tukey’s Honestly Significant Difference test were used for multiple comparisons (different numbers of “*” denoted by p < 0.05). All statistical analyses were performed using GraphPad Prism 8.0 software.

## 3 Results

### 3.1 ACP-associated virus and *C*Las screening with RT-PCR in individual insects

Using the RNA in adults of ACPs as a template and the primers listed in **Table 1**, the target gene fragments of six pathogens, DcRV, DcPLV, DcBV, DcDV, *C*Las, and DcBV2, and the internal reference gene *β-Actin* were successfully detected (**Supplementary Figure S1**). The blasting results in the NCBI database showed more than 98% similarity, indicating that the target fragments of each pathogen and internal reference gene were successfully amplified.

**Figure 1:**
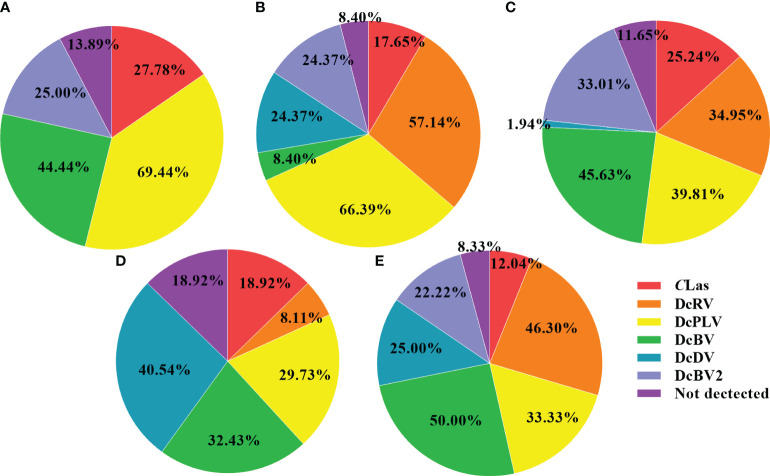
Pie chart showing the percentage of *C*Las and five viruses detected in different regions. **(A)**, Guizhou; **(B)**, Guangdong; **(C)**, Guangxi; **(D)**, Jiangxi; **(E)**, Sichuan. Note that in the majority of the five regions of China, there were multiple samples that had multiple pathogens, therefore, most of the percentages in the pie charts do not equal 100%. *C*Las, *Candidatus* Liberibacter asiaticus; diaphorina citri reovirus, DcRV; diaphorina citri picorna-like virus, DcPLV; diaphorina citri bunyavirus, DcBV; diaphorina citri densovirus-like virus, DcDV; diaphorina citri bunyavirus 2, DcBV2.

ACPs were collected from Guizhou, Guangdong, Guangxi, Sichuan, and Jiangxi in southern China to detect infection by *C*Las and 5 viruses in individual insects. As shown in **Table 2**, **Figure 1**, the detection rate of DcPLV was 55.35% in all ACP samples. In this study, 551 ACPs were collected, 305 of which had DcPLV. The detection rate of DcPLV was highest in ACPs in Guizhou and Guangdong, with detection rates of 69.44% and 66.39%, respectively. The detection rate of DcRV was also high in Guangdong and Sichuan ACPs (57.14% and 46.30%, respectively), while it was 28.49% in all samples. However, DcRV and DcDV were not detected in ACPs from Guizhou. DcBV had a slightly higher detection rate than DcBV2, both of which belong to *Bunyavirales*. In addition, the infection rate of *C*Las in all ACPs was 19.78%.

**Table 2:** Total number and detection percentages of CLas and Asian citrus psyllid (ACP)-associated viruses in various surveyed regions.

Survey regions	Total numbers of samples	Number of infected samples					
		*C*Las	DcRV	DcPLV	DcBV	DcDV	DcBV2
Guizhou	108	30	0	75	48	0	27
Guangdong	119	21	68	79	10	29	29
Guangxi	103	26	36	41	47	2	34
Sichuan	108	13	50	36	54	27	24
Jiangxi	113	19	3	74	70	18	10
Total	551	109	157	305	229	76	124
Infection rate (%)		19.78	28.49	55.35	41.56	13.79	22.50

CLas, Candidatus Liberibacter asiaticus; diaphorina citri reovirus, DcRV; diaphorina citri picorna-like virus, DcPLV; diaphorina citri bunyavirus, DcBV; diaphorina citri densovirus-like virus, DcDV; diaphorina citri bunyavirus 2, DcBV2.

### 3.2 Co-infection of *C*Las and 5 ACP-associated viruses in individual ACPs

Among all evaluated ACPs, 61.16% were co-infected with multiple symbionts, as shown in **Table 3**. Of the combinations of two symbionts co-infecting ACPs, DcPLV+DcBV had the highest co-infection rate, reaching 10.16% of all samples, followed by *C*Las+DcPLV with 4.17%. Among the co-infections involving three pathogens, DcRV+DcPLV+DcBV had the highest infection rate (3.45%), followed by *C*Las+DcPLV+DcBV (3.27%). In addition, the infection rate of ACPs infected with more than 4 pathogens was 4.36%.

**Table 3:** Co-infection of *C*Las and 5 viruses in Asian citrus psyllid (ACP) samples.

Co-infection type	Number	Co-infection rate (%)
*C*Las+DcRV	6	1.09
*C*Las+DcPLV	23	4.17
*C*Las+DcBV	7	1.27
*C*Las+DcDV	2	0.36
*C*Las+DcBV2	3	0.54
DcRV+DcPLV	19	3.45
DcRV+DcBV	21	3.81
DcRV+DcDV	6	1.09
DcRV+DcBV2	16	2.90
DcPLV+DcBV	56	10.16
DcPLV+DcDV	9	1.63
DcPLV+DcBV2	19	3.45
DcBV+DcDV	5	0.91
DcBV+DcBV2	10	1.81
DcDV+DcBV2	2	0.36
*C*Las+DcRV+DcPLV	4	0.73
*C*Las+DcRV+DcBV	2	0.36
*C*Las+DcRV+DcDV	1	0.18
*C*Las+DcRV+DcBV2	2	0.36
*C*Las+DcPLV+DcBV	18	3.27
*C*Las+DcPLV+DcDV	3	0.54
*C*Las+DcPLV+DcBV2	3	0.54
*C*Las+DcBV+DcDV	1	0.18
*C*Las+DcBV+DcBV2	5	0.91
*C*Las+DcDV+DcBV2	1	0.18
DcRV+DcPLV+DcBV	19	3.45
DcRV+DcPLV+DcDV	9	1.63
DcRV+DcPLV+DcBV2	7	1.27
DcRV+DcBV+DcDV	2	0.36
DcRV+DcBV+DcBV2	6	1.09
DcRV+DcDV+DcBV2	1	0.18
DcPLV+DcBV+DcDV	7	1.27
DcPLV+DcBV+DcBV2	16	2.90
DcPLV+DcDV+DcBV2	1	0.18
DcBV+DcDV+DcBV2	1	0.18
Other	24	4.36
Total	337	61.16

Co-infection rate = Number of co-infected ACPs/total number of ACPs, CLas, Candidatus Liberibacter asiaticus; diaphorina citri reovirus, DcRV; diaphorina citri picorna-like virus, DcPLV; diaphorina citri bunyavirus, DcBV; diaphorina citri densovirus-like virus, DcDV; diaphorina citri bunyavirus 2, DcBV2.

**Figure 2:**
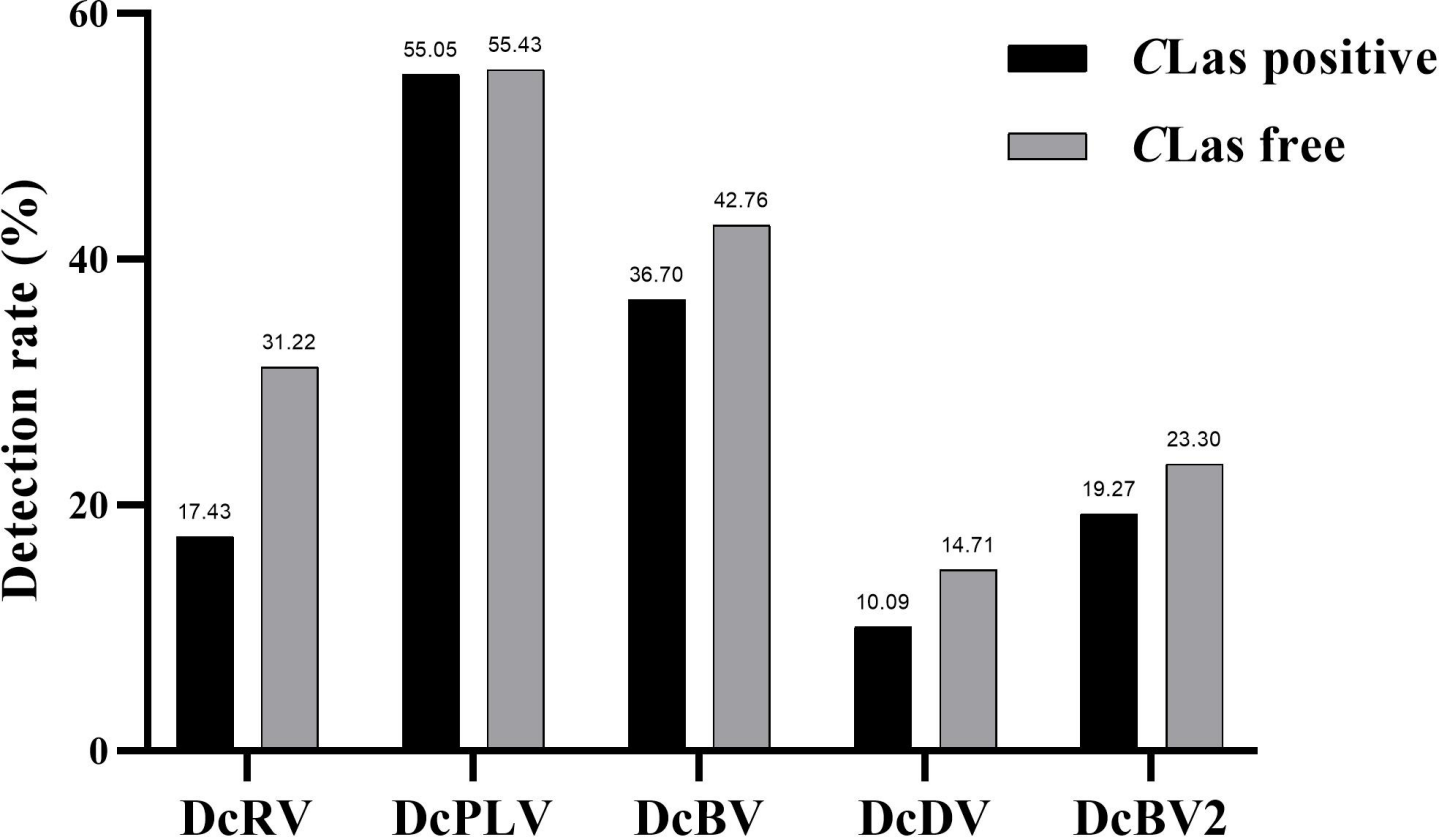
Detection rate of Asian citrus psyllid (ACP)-associated viruses in *Candidatus* Liberibacter asiaticus (*C*Las)-positive and *C*Las-free ACP samples. diaphorina citri reovirus, DcRV; diaphorina citri picorna-like virus, DcPLV; diaphorina citri bunyavirus, DcBV; diaphorina citri densovirus-like virus, DcDV; diaphorina citri bunyavirus 2, DcBV2.

**Figure 3:**
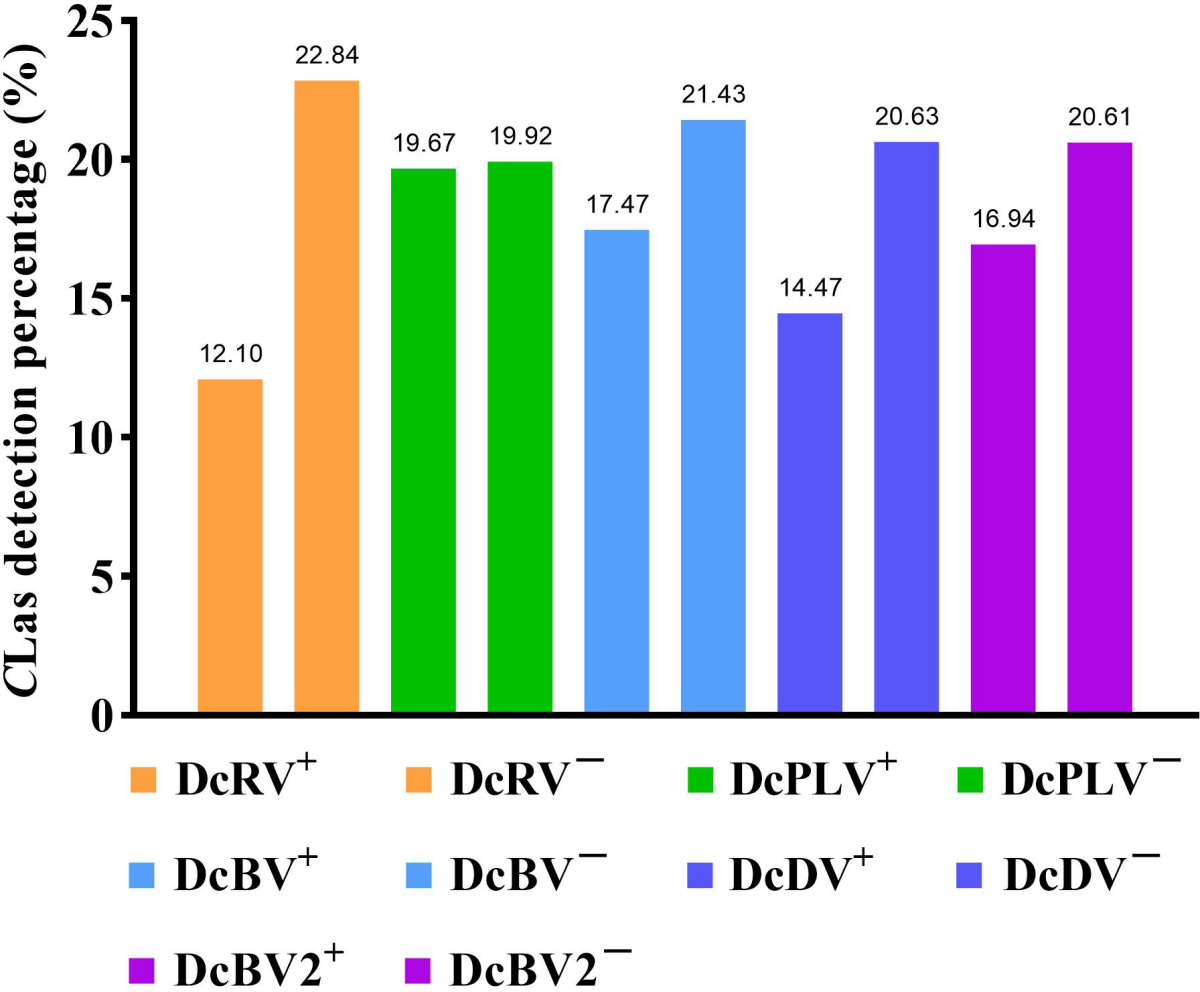
Detection percentage of *Candidatus* Liberibacter asiaticus (CLas) in Asian citrus psyllid (ACP)-associated virus-positive and virus-free ACP samples. +: ACP-associated virus-infected, −: ACP-associated virus-uninfected. diaphorina citri reovirus, DcRV; diaphorina citri picorna-like virus, DcPLV; diaphorina citri bunyavirus, DcBV; diaphorina citri densovirus-like virus, DcDV; diaphorina citri bunyavirus 2, DcBV2.

Among 109 *C*Las-positive ACPs, 83.49% were co-infected with one or more ACP-associated virus (data not shown). The infection rates of DcRV, DcDV, DcBV and DcBV2 in *C*Las-positive samples were lower than those in *C*Las-free ACPs, especially for DcRV. The detection rate of DcRV was close to 50% lower in *C*Las-positive ACPs than in *C*Las-free ACPs (**Figure 2**). A similar result was seen for the detection of *C*Las in DcRV-infected ACP (**Figure 3**). No decrease in the detection of DcPLV was observed between *C*Las-positive and *C*Las-free ACPs (**Figure 2**). A similar result was observed for *C*Las detection in DcPLV- infected ACPs (**Figure 3**).

### 3.3 Distribution and transmission of DcPLV in ACPs

**Figure 4:**
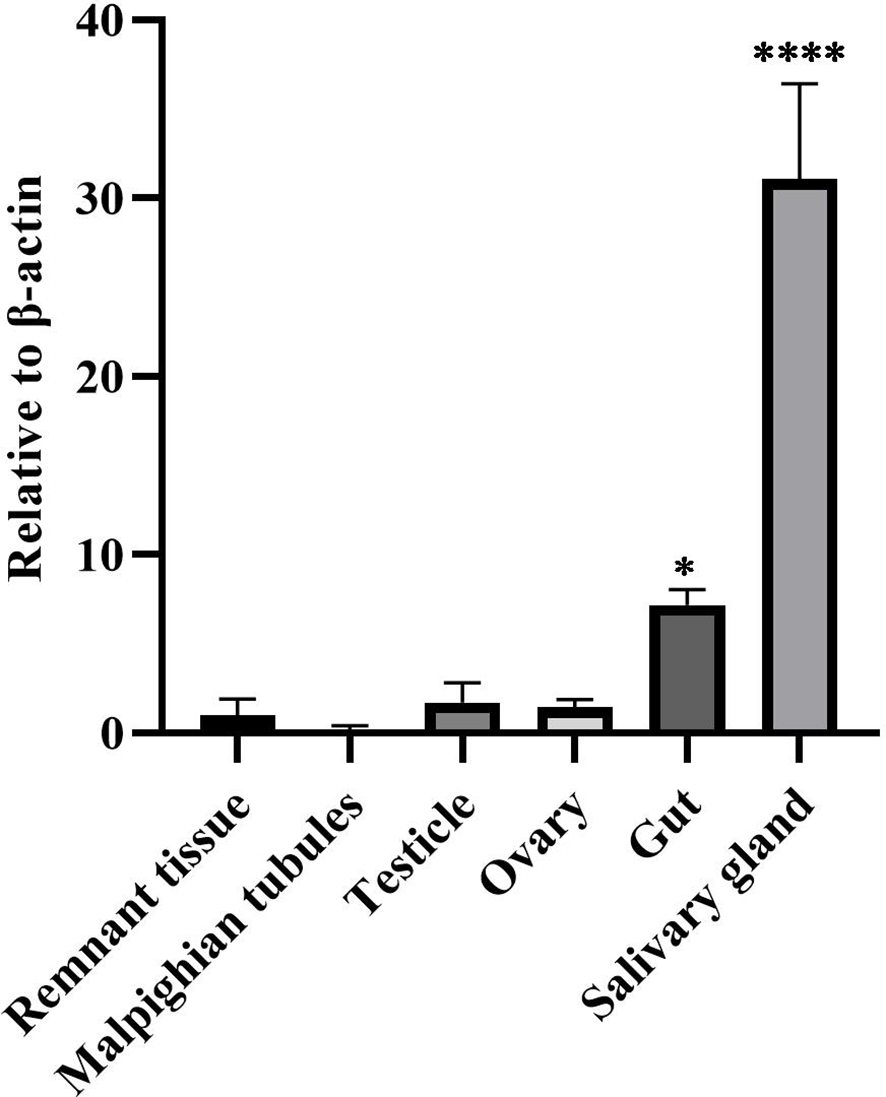
Relative contents of diaphorina citri picorna-like virus (DcPLV) in different tissues of Asian citrus psyllid (ACP). Data are means (± SE) of four biological replications per sample; *β-Actin* was used to normalize the expression of DcPLV as the reference gene. One-way ANOVA followed by the Tukey’s Honestly Significant Difference test were used for multiple comparisons (different numbers of “*” denoted by p < 0.05). The greater the number of *, the greater the variability.

To understand the distribution of DcPLV in different organs of ACPs, we assessed the levels of DcPLV in the guts, testicles, ovaries, salivary glands, Malpighian tubules, and remnant tissues. RT-qPCR results showed that DcPLV was highest in salivary glands among the 5 tissues. In Malpighian tubules, we hardly detected DcPLV (**Figure 4**).

We collected 56 offspring of ACPs, 22 of which carried DcPLV. The proportion of DcPLV-positive offspring was 39.29%. We also performed RNA testing on the leaves of orange jessamine that ACPs fed on and determined the presence of DcPLV. However, we did not identify DcPLV in the leaves that ACPs did not feed on at the same time as orange jessamine.

## 4 Discussion

HLB is a major threat to the citrus industry. Its vector, ACP, is also a key control target. ACPs not only carry *C*Las but are also hosts for many insect viruses. Prior to this study, Britt et al. (2020) investigated insect viruses in ACPs in Florida with five ACPs as a group sample and found that the most prevalent virus was DcACV. To understand the prevalence of some insect viruses and *C*Las in ACPs in China, this study collected ACP samples from the five main citrus-producing regions in China, Guizhou, Jiangxi, Guangdong, Guangxi, and Sichuan, and detected DcRV, DcPLV, DcBV, DcDV, *C*Las, and the newly discovered virus DcBV2 in individual ACPs using RT-PCR. DcPLV was the most prevalent virus in the collected ACP samples in China in this study. Regional differences may result in these population differences. In addition, we attempted to detect two previously identified viruses, DcACV and DcFLV. However, positive ACP samples were not found for these viruses. More samples may be needed to determine whether the two viruses are present. Chen et al. (2020) investigated the infection of ACPs with DcRV in Fuzhou, Fujian, China, and their incidence rate was 58-100%, similar to the detection rates of DcRV in Guangdong and Sichuan. However, the detection rates of DcRV in ACPs in other regions were relatively low. This suggests that the virus prevalence varies among countries and regions. To our knowledge, this is the first time that a comprehensive survey has been conducted in China to detect the prevalence of these viruses in individual ACPs. In addition, the detection rate of *C*Las was lowest in ACPs in Sichuan, at 12.04%, which is similar to the detection rate of *C*Las in citrus samples from Sichuan province (Cui et al., 2022).

Studies have shown that some insect viruses may affect host adaptation and reproduction (Vasilakis and Tesh, 2015). For instance, ABV-1 reduced the total aphid nymphal duration and induced reproduction (An et al., 2022). The aphid-lethal paralysis virus affects the movement and lifespan of *Rhopalosiphum padi* (Williamson et al., 1988). Zhang et al. (2022) found that crude extract containing seven endosymbiotic viruses, including DcRV and DcPLV, could significantly reduce the total egg production of female populations after injection into ACPs. However, whether the 5 viruses (DcRV, DcPLV, DcBV, DcDV, and DcBV2) play a role is not clear. In this study, DcPLV had the highest detection rate among the ACPs. DcPLV has been suggested as a new and unclassified picorna-like virus, although a phylogenetic tree generated based on the RdRp placed DcPLV close to *Iflaviridae* (Nouri et al., 2016). The picorna-like viruses, with a close taxonomic relationship to DcPLV, are also present in the wild lime psyllid and the potato psyllid (Stuehler et al., 2023). Because of this, we need to have some understanding of DcPLV. According to our survey, DcPLV is not only capable of propagating vertically but also has the potential to propagate horizontally. However, this does not mean that DcPLV can infect plants; it was not found in the leaves of the same plant not fed on by ACP. DcPLV was most prevalent in the salivary glands, indicating that it may spread horizontally. The picorna-like deformed wing virus has been associated with wing deformities in adult honeybees. It can infect the various developmental stages of bees (Mcmenamin and Flenniken, 2018). However, another picorna-like virus, helicoverpa armigera Nora virus (HaNV), has been shown to be efficiently horizontally transmitted between hosts via contaminated food and transmitted vertically from parent to offspring. Moreover, HaNV is not overtly pathogenic to its host (Yang et al., 2019). The high prevalence of DcPLV in China’s ACP populations indicates that it might be well adapted to ACPs. However, whether DcPLV can affect the growth habits of ACPs is unclear. It is unknown whether DcPLV has the potential to be used as a genetically engineered viral candidate to control insect vectors using RNA interference technology. Therefore, more studies on DcPLV as a virus vector are needed.

Previous studies have shown that the interaction between different pathogens in insect vectors may be antagonistic or cooperative (Domingo-Calap et al., 2020). The acquisition or transmission of one symbiont in insects can be affected by another symbiont. The *Mal de Río Cuarto virus* titer was reduced after its planthopper vector was co-infected with a wheat rhabdovirus (Dumon et al., 2018). Glaser and Meola (2010) suggested that the infection of *Wolbachia* in vector insects can induce resistance to the *West Nile virus*. The commensal bacterium *Sulcia* can interact with the *Rice dwarf virus* to co-localize to oocytes, helping the virus spread vertically through the egg to the offspring of leafhoppers (Wu et al., 2019). Moreover, *C*Las has been found to affect the endosymbiont abundance of ACPs using high-throughput metagenome sequencing technology (Pan et al., 2023). In the present study, *C*Las and four insect viruses (DcRV, DcDV, DcBV and DcBV2), especially DcRV, are prone to mutually reduced detection in the ACP population in the field. The number of ACPs co-infected with CLas and DcRV decreased by about 50% compared to those infected with only one pathogen, indicating that co-infection had a negative effect on the insect host. DcRV, a new species of the genus *Fijivirus*, is a persistent infection in its psyllid host and is distributed throughout the bodies of ACPs, including the gut and salivary glands (Chen et al., 2019). In addition, as *C*Las has a circulative–propagative transmission cycle in psyllids, *C*Las is associated with the gut, hemolymph, salivary glands, and fat bodies (Glaser and Meola, 2010; Ammar et al., 2011). *C*Las induces changes in different pathways including the insect’s metabolism and immune system (Kruse et al., 2017). Because of the similar distribution, *C*Las and DcRV could compete for resources within ACP, such as the metabolism of amino acids. *C*Las and DcRV could also alter similar host-immune responses. Our results were just analyzed based on infection from ACP in the field. More studies need to be performed on the specific change in *C*Las content with DcRV and other viruses in ACP under strict experimental control.

## 5 Conclusion

The infection rates of *C*Las and five insect viruses in ACP samples from five main citrus-producing regions in China were analyzed. DcPLV was the most prevalent and widespread ACP-associated virus. DcPLV was also demonstrated to propagate vertically and found more in salivary glands among different tissues. Approximately 60% of adult insect samples were co-infected with more than one insect pathogen. These results provide valuable information about the prevalence of ACP-associated viruses in China. In addition, analysis of the change in endosymbiont infection in a single insect vector is the basis for understanding the interaction between *C*Las, ACP, and insect viruses.

## Data availability statement

The raw data supporting the conclusions of this article will be made available by the authors, without undue reservation.

## Ethics statement

The manuscript presents research on animals that do not require ethical approval for their study.

## Author contributions

LL: Data curation, Formal analysis, Investigation, Writing – original draft, Writing – review & editing. JC: Data curation, Investigation, Writing – review & editing. JJ: Investigation, Software, Writing – review & editing. JML: Data curation, Validation, Writing – review & editing. YS: Data curation, Validation, Writing – review & editing. QC: Data curation, Methodology, Writing – review & editing. FY: Data curation, Methodology, Writing – review & editing. ZB: Writing – review & editing. ZS: Conceptualization, Writing – review & editing. JXL: Project administration, Validation, Data curation, Conceptualization, Writing – original draft, Writing – review & editing.

## Supplementary material

Supplementary Figure 1 RT-PCR amplification and verification of 5 Asian citrus psyllid associated viruses, *C*Las and *β-Actin* by 1.2% agarose gel electrophoresis. *C*Las, *Candidatus* Liberibacter asiaticus; diaphorina citri reovirus, DcRV; diaphorina citri picorna-like virus, DcPLV; diaphorina citri bunyavirus, DcBV; diaphorina citri densovirus-like virus, DcDV; diaphorina citri bunyavirus 2, DcBV2.
