# Supplementary material for: Detection of Candidatus Liberibacter asiaticus and five viruses in individual Asian citrus psyllid in China
Source: Front Plant Sci. 2024 Feb 6;15:1357163. doi: 10.3389/fpls.2024.1357163 (PMC10877018; doi:10.3389/fpls.2024.1357163)
Supplement: Supplementary file 1 [file Table_1.docx]

**Table S1** Locations and quantities of Asian citrus psyllid collected.

| Province | City/Country | Number |
| --- | --- | --- |
| Guizhou | Luodian | 108 |
| Guangdong | Guangzhou | 85 |
|  | Sihui | 34 |
| Guangxi | Guilin | 103 |
| Sichuan | Pingshan | 12 |
|  | Leibo | 96 |
| Jiangxi | Zhanggong | 37 |
|  | Ganxian | 33 |
|  | Xinyv | 19 |
|  | Huichang | 24 |
