# Supplementary material for: Detection of Candidatus Liberibacter asiaticus and five viruses in individual Asian citrus psyllid in China
Source: Front Plant Sci. 2024 Feb 6;15:1357163. doi: 10.3389/fpls.2024.1357163 (PMC10877018; doi:10.3389/fpls.2024.1357163)
Supplement: Supplementary file 2 [file Table_2.docx]

**Table S2** The concentration of RNA isolated from various organs of Asian citrus psyllid.

| Organ | RNA concentration (ng/μL) |
| --- | --- |
| Guts 1 | 120.3 |
| Guts 2 | 231 |
| Guts 3 | 163 |
| Guts 4 | 340.7 |
| Salivary glands 1 | 109.3 |
| Salivary glands 2 | 153.2 |
| Salivary glands 3 | 102.4 |
| Salivary glands 4 | 300 |
| Testes 1 | 200 |
| Testes 2 | 377.1 |
| Testes 3 | 235.4 |
| Testes 4 | 430.6 |
| Ovaries 1 | 205.2 |
| Ovaries 2 | 206.9 |
| Ovaries 3 | 172.8 |
| Ovaries 4 | 459.4 |
| Malpighian tubules 1 | 123.5 |
| Malpighian tubules 2 | 112.2 |
| Malpighian tubules 3 | 128.4 |
| Malpighian tubules 4 | 199.9 |
| Remnant tissues 1 | 449.7 |
| Remnant tissues 2 | 341.2 |
| Remnant tissues 3 | 482.2 |
| Remnant tissues 4 | 783.2 |

RNA was solubilized with 20 μL RNase-free Water. The concentration of RNA was measured by Nanodrop 2000 (Thermo Scientific, Hongkong, China).
