# Supplementary figures and images for: Detection of Candidatus Liberibacter asiaticus and five viruses in individual Asian citrus psyllid in China
Source: Front Plant Sci. 2024 Feb 6;15:1357163. doi: 10.3389/fpls.2024.1357163 (PMC10877018; doi:10.3389/fpls.2024.1357163)

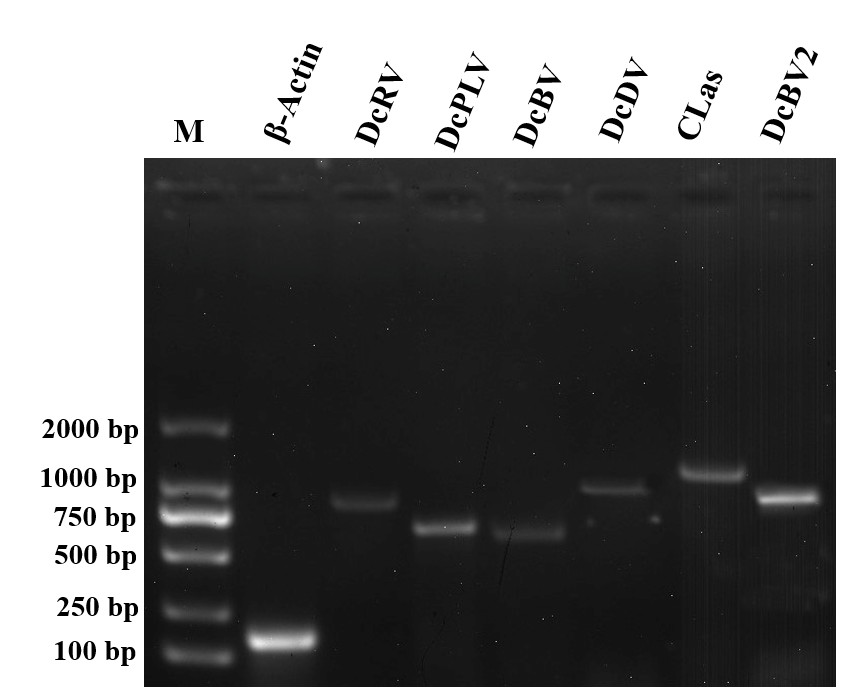

Supplement: Supplementary Figure 1 — RT-PCR amplification and verification of 5 Asian citrus psyllid associated viruses, CLas and β-Actin by 1.2% agarose gel electrophoresis. CLas, Candidatus Liberibacter asiaticus; diaphorina citri reovirus, DcRV; diaphorina citri picorna-like virus, DcPLV; diaphorina citri bunyavirus, DcBV; diaphorina citri densovirus-like virus, DcDV; diaphorina citri bunyavirus 2, DcBV2. [file Image_1.tif]
